# Tourism marketing in the metaverse: A systematic literature review, building blocks, and future research directions

**DOI:** 10.1371/journal.pone.0300599

**Published:** 2024-05-10

**Authors:** Eva Sánchez-Amboage, Verónica Crespo-Pereira, Matías Membiela-Pollán, João Paulo Jesús Faustino

**Affiliations:** 1 Business Department, University of A Coruña, A Coruña, Spain; 2 Faculdade de Letras, University of Porto, Porto, Portugal; University of Pisa, ITALY

## Abstract

The aim of this research is to investigate tourist marketing within the embryonic context of the metaverse in order to comprehend the building blocks and the primary technologies employed in the sector. A systematic literature review (SLR) was conducted on 386 articles, with an overall qualitative approach that included 86 references, all of which dealt with the topic of the metaverse and had direct or potential implications for the tourism sector (hotels, restaurants, means of transport, leisure activities and destination itself). The articles are taken from: Science Direct, Taylor & Francis, Emerald, Springer and Google Scholar. The SLR was carried out according to the PRISMA search protocol. The results indicate the technologies that have been most thoroughly studied at the confluence of marketing, tourism, and the metaverse (AI, virtual reality, augmented reality, mixed reality, blockchain, tokens (NFTs) and digital twins). Moreover, they establish the foundational components of tourism marketing in the metaverse for the first time (tourism products, the metaverse as a distribution and branding channel for tourism and, tourist customer as protagonist). Finally, the study exposes research gaps and recommends future directions for exploration (monetization of products in the metaverse, promotion and marketing strategies in the metaverse, new profiles for marketing professionals, policy development that regulates commercial activity in the metaverse).

## Introduction

The COVID-19 pandemic changed how societies and economies developed around the world [1]. No other previous global crisis has affected every country and every industry [2]. In addition to economic losses, the quarantine and social isolation have been detrimental to our social and psychological well-being [3]. At the root of all this, the contactless culture has been firmly established in society and in our daily lives [4]. Tourists have exhibited new behaviors, such as taking precautionary health measures when traveling or avoiding crowded places, events and/or group travel, for example. We are faced with a “new tourist” who demands touchless/contactless travel [5] that matches their lifestyle, consisting of leisure, remote work, family obligations and hybrid activities (both virtual and real) [6].

Technology is serving as a vector of change in this post-COVID society. In the tourism industry, technological innovation is playing a fundamental role in the post-pandemic recovery [7]. Without information technology (IT), there would have been no tourism during and after COVID-19 [8]. The traditional service experience is changing "high-touch and low-tech” processes into “low-touch and high-tech” ones [9]. For example, in the hotel sector, technology has made it possible to reduce interactions between customers and staff through contactless check-in and check-out systems, digital key systems, face recognition systems, cleaning robot systems [7], as well as creating new promotion options through livestreaming [10]. Furthermore, during the main quarantine period, several tourism services and activities changed from on-site to totally digital and virtual formats. Major brands opted to reformulate communication and digital marketing strategies to boost interaction with their audiences. Companies like Airbnb created an “online experience” section [11]; restaurants have adopted new measures to maintain their income and retain employment levels [12]; museums around the world conducted live visits, primarily through the social media [8] and tourist destinations shared their history and areas of interest over the Internet, with the main goal being to connect with future tourists at an extremely complicated time worldwide. The pandemic has triggered and accelerated change [13,14], however, these practices were already latent in the tourism industry even before the pandemic [15].

This is where metaverse comes into play, an interconnected ecosystem of digital and physical environments that can be experienced simultaneously, seamlessly blending physical and technological realities [16]. The concept of the metaverse and the virtual experiences related to it have emerged in society and have radically changed the future of technology and its potential impact on the hospitality and tourism industry [6,17,18].

Although, the metaverse is positioned as one of the most popular research agendas [19], only two articles related to tourism and the metaverse have been published in specialized tourism journals until 2022: [20,21]. Authors such as [22] understand that the metaverse will be the marketing platform of the future, where communication with customers will be different from what we know now. [6,22] discuss the foundations and building blocks for marketing in the metaverse, while [23] consider the building blocks of tourism in the metaverse. There are no references to the building blocks of tourism marketing in the metaverse.

This systematic literature review (SLR) was conducted to address these gaps, to expand the framework for tourism marketing in the metaverse, and to identify areas for future research.

This paper presents a systematic literature review (SLR) of academic publications related to the metaverse that have direct or potential impact on tourism. The aim of this research is to investigate tourist marketing within the embryonic context of the metaverse in order to comprehend the building blocks and the primary technologies employed in the sector.

The results obtained from both objectives can be employed in other research areas within the creative industries. Across various sectors, companies share common characteristics. Those within the creative industries particularly emphasize the creation of original and innovative content, spanning products, services, or experiences. Creativity and originality serve as foundational values in these enterprises. Examples of businesses in the creative industry encompass various areas such as visual arts, traditional culture, cultural sites, publications, new media, etc.

Preliminary metaverse studies will be able to share their findings to create knowledge about the metaverse marketing discipline.

Next, the research is structured into three sections: methodology, which provides a detailed explanation of the systematic literature review; results, focusing on the most studied metaverse technologies in tourism research and the building blocks of tourist marketing in the metaverse. Finally, the research concludes with a section on conclusions, limitations, and future research directions.

## Methodology

Review works are widely accepted in the academic field. Since 2012, journals focused on tourism have increased the number of review articles published, which reflects the growing popularity of this type of studies. In terms of their repercussion, the review articles most frequently cited by other authors fall under the topics of economics and finance and marketing [24].

Within review works, systematic reviews aim to summarize and analyze evidence with regard to an objective or research question. Systematic reviews are based on specifying the method used to find, select, analyze and synthesize the primary sources used in the research [25].

The present research is conducted considering the Preferred Reporting Items for Systematic Reviews and Meta-Analyses (PRISMA) search protocol [26].

**Fig 1:**
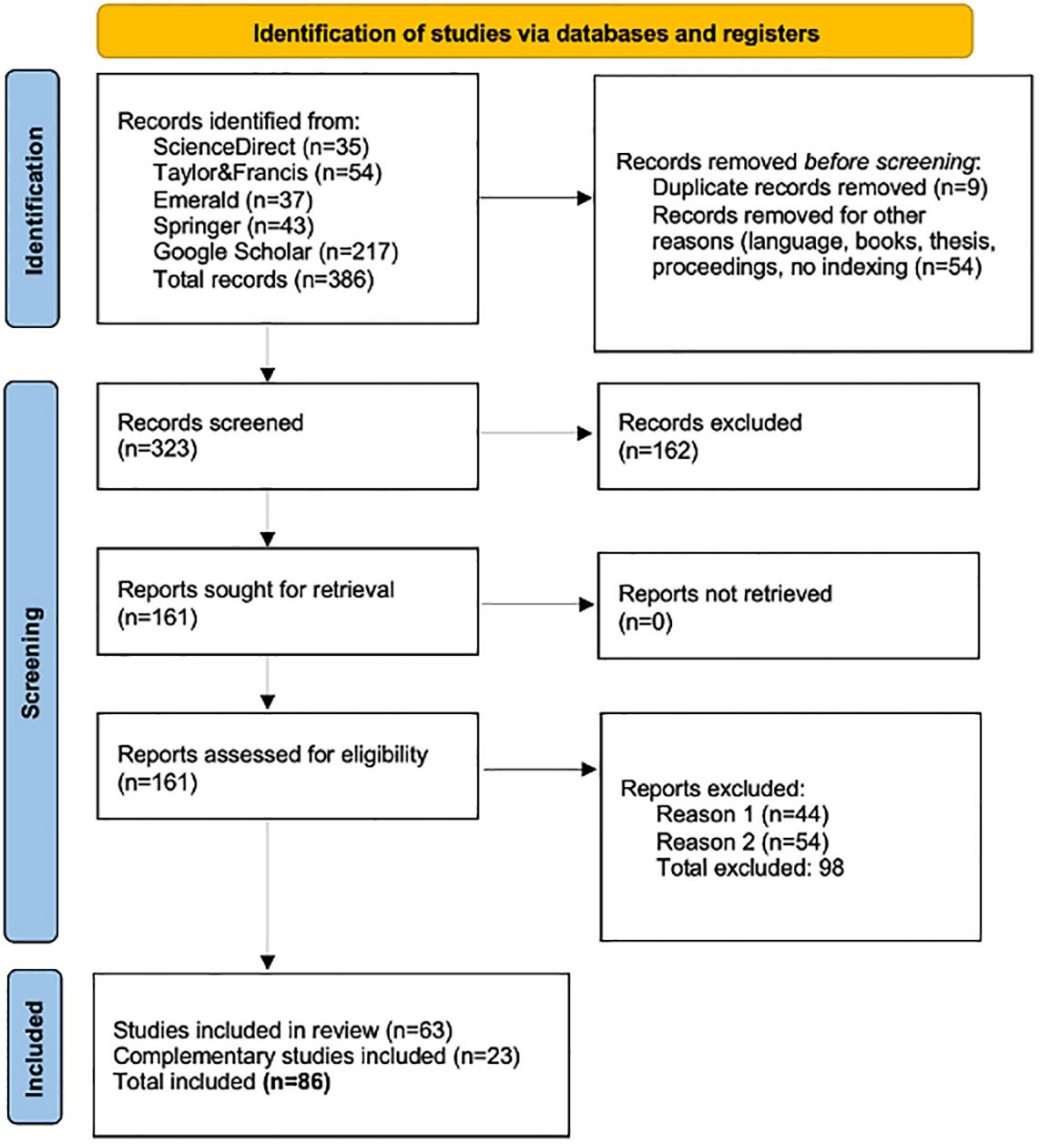
PRISMA flow chart process of article selection (adapted from [28]).

PRISMA is a protocol for conducting systematic reviews that consists of a 27-item checklist and a four-phase flow chart (Fig 1). It was developed in the field of medicine by a group of 29 scholars with the intention of increasing the transparency and precision of literature reviews. The reason for choosing PRISMA over other existing protocols lies in its recognition and use by various disciplines throughout the world beyond the medical fields, as well as its potential for improving the validity and confidence of the systematic reviews in hospitality services and tourism [27].

The prior publication of the protocol reduces the impact of inherent biases by the author and promotes transparency about the methods and process, as well as preventing redundant reviews. Among the many options that exist for evaluating bias risk, PRISMA promotes a system based on the evaluation of different key design components and the execution of studies so that there is solid empirical evidence of its relationship to the bias [29].

### Eligibility criteria and search strategy

Several searches in different databases were carried out to decide which were the most pertinent, in terms of the number of articles revealed and the affinity of these articles to the proposed research topic. The search equation that ultimately presented the most beneficial search results was: marketing AND (metaverse OR "metaverse platform") AND (tourism OR travel OR hospitality).

It was decided to perform the search on the collections of publications most used in the fields of research on marketing, hospitality services and tourism: Science Direct, Taylor & Francis, Emerald and Springer. This search was complemented by the results from the Google Scholar search engine, since in recent years it has significantly expanded its coverage [30]. The filters used in the search of publication collections and Google Scholar were: scientific articles, English language and any date. Those references that come from non-indexed journals are eliminated. The research has examined articles on marketing and the metaverse, with direct or indirect relevance to the tourism sector, from 1992 to 2022, with a specific focus on the years 2020–2022 due to the significance of these years of publication, as explained later in "reports excluded, reason 1".

The Scopus and Web of Science databases were ruled out as they contain a small number of articles for analysis, 2 and 6, respectively; and for these not being totally related to the research objective. The same search equation was used in Proquest, obtaining a total of 293 records (doctoral theses), which after the analysis of the title, abstract and key words demonstrated that the results were not closely linked to the research proposal and thus this database was also ruled out.

### Screening

An Excel document was developed to save the results, organized based on: code, title, author, key words, abstract, year, journal, DOI and origin of the corresponding author. The document has been registered on Zenodo and can be accessed through the following link: https://doi.org/10.5281/zenodo.10782765

The “code” cell helps structure the study selection process as follows and reduces the risk of researcher bias. The inclusion or exclusion of each reference has been validated by all four authors of this article.

- *Records excluded*: (n = 162) reviewing the title, abstract, key words and determining that the topic does not match: metaverse, blockchain, XR, AR, VR, second life, IA, virtual world.
- *Reports excluded*, *reason 1*: (n = 44) articles prior to 2020. No filter is used with regard to the search date (“anytime”). However, after the first analyses, it was decided that the studies that would form part of the SLR would be those that were published in the last two years (2020–2022). Authors like Kim (2021) explain that the term metaverse has gained ground in the world of technology since 2020, becoming popular since 2021 when it coincided with the change of the Facebook brand to Meta, among other events.
- *Reports excluded*, *reason 2*: (n = 54) lack of agreement with the topic. These are articles that include the word metaverse, but are not considered to fall under the social sciences (e.g. the field of medicine), or articles that address the topic but do not offer pertinent information for our research.
- *Reports Included*, *reason 1*: (n = 10) articles that coincided with the study topic: tourism, metaverse, marketing. Also considered were those articles that, in spite of not including the word metaverse, second life or virtual word, deal with their technologies: blockchain, XR, AR, VR, IA, IoT and NFT.
- *Reports Included*, *reason 2*: (n = 53) articles that deal with marketing and the metaverse, but that are not focused on the tourism industry, however, their information can potentially be applied in the field of tourism.

As the SLR progressed and due to the scarce number of references, it became necessary to include articles that are complementary to the research. These are articles that have been references in the SLR articles, using the “snowball” strategy.

- *Reports Included*, *reason 3*: (n = 14) articles from the complementary search that coincide with the study topic: tourism, metaverse, marketing.
- *Reports Included*, *reason 4*: (n = 9) articles from the complementary search on marketing and the metaverse from other sectors, with an implication for tourism.

In complementary research there are four articles that date back to before the year 2020. The inclusion of these articles poses an important bias risk. In order to avoid this, each reference has had to pass a review by all four authors.

*Search* *protocol* *registration*

- A search protocol registration has been developed for research on OSF registries.
- Registration name: tourism marketing in the metaverse: a systematic review
- Registration type: OSF Preregistration
- Registration DOI: https://doi.org/10.17605/OSF.IO/B9V75

## Results

### SLR general statistics

The findings from this review have indicated that the articles focused on the tourism sector and the metaverse are few (n = 24) (reports included reason 1 (n = 10) and reports included reason 3 (n = 14)), which indicates that for the time being, the topic of study is novel and more knowledge on the subject is needed.

**Fig 2:**
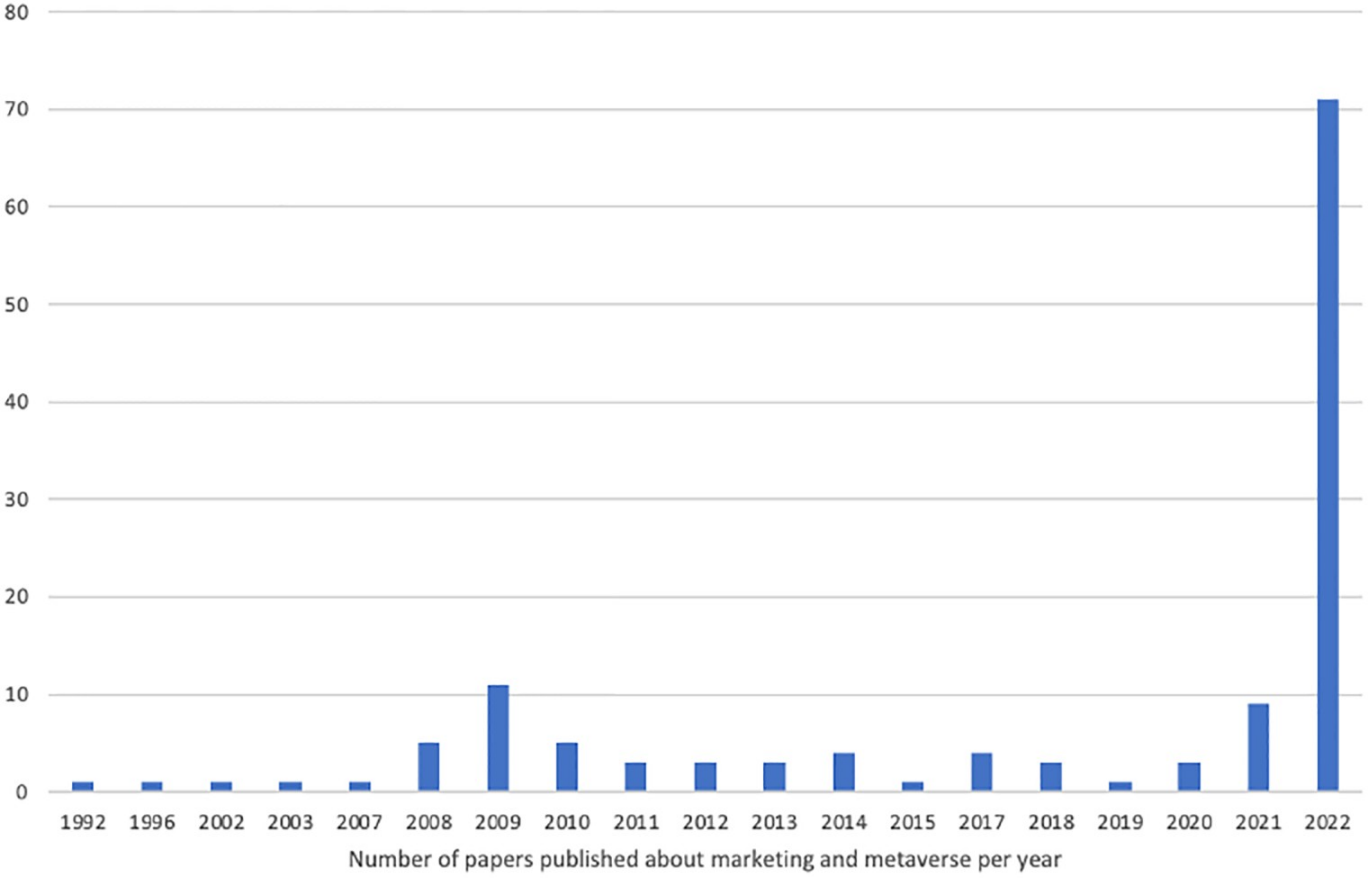
Total number of papers published about marketing and metaverse per year.

Publications on marketing and the metaverse have been fairly recent. Most of them have been concentrated in the year 2022 (Fig 2). Through screening the SLR, it was observed that the main themes about the metaverse evolve over time. Articles published before 2020 focus on topics such as Second Life, virtual world, and 3D, while articles after 2020 cover topics like metaverse, blockchain, XR, AR, VR, Second Life, AI, virtual world, IoT, and NFTs.

Table 1 shows that, 43% of the articles on the metaverse analyzed are from European universities, primarily the United Kingdom (12) and Germany (4). The topic is also studied in Asia (29%), notably in universities in South Korea (8) and China (7). The United States appears as the leading country in research in this area, with 11 publications. These statistics show that the metaverse is a topic of global interest, with research efforts concentrated in Europe, Asia, and the United States. This distribution reflects the widespread curiosity and exploration of the metaverse concept across different regions and academic institutions.

**Table 1:** Country of origin of the reports included in the research (n = 86).

Continents	Countries	Number of researches
America	USA (11), Canada (2), Brazil (1), Trinidad & Tobago (1)	15
Europe	UK (12), Germany (4), The Netherlands (2), Sweden (2) Slovak republic (2), Norway (3), Finland (2), Spain (2), Switzerland (1), Turkey (3), Slovenia (1), Ireland (1), Cyprus (1), Serbia (1)	37
Africa	South Africa (2), Nigeria (1)	3
Asia	Irán (3), United Arab Emirates (1), Qatar (1), Kingdom of Saudi Arabia (1), South Korea (8) China (7), India (2), Malaysia (2), Tailandia (1), Taiwan (1), Brunei (1), Bangladesh (1)	29
Oceania	Australia (2)	2

* The country of origin is taken from the address of the first corresponding author of each article.

### Metaverse concept

Most of the studies analyzed agree that the word metaverse is not a recent one, as it was referenced in the science fiction novel “Snow Crash” by Neal Stephenson in 1992 [31]. Even [20] remind us that the concept could date back as far as 1909, since it is also mentioned in the work “Machine Stops” by E. M. Foster. In any case, its popularity has grown with the release of Second Life in 2003 [22], only to take off in 2021, supported by a more developed technological scenario and after different brands began to propose their activity in the metaverse (e.g. Facebook rebrand itself as Meta) [32]. Several articles analyzed also expose the idea that the term Metaverse combines “meta” (meaning post, after or beyond) and “verse” (universe) [4,6,33] and it is defined as an ecosystem of shared and interconnected digital and physical environments that can be experienced in a synchronous manner, where physical and technological realities are seamlessly combined [16]. Enabled by Internet 3.0, the metaverse refers to a three-dimensional virtual space that focuses on social connections [20] or in a reductionist definition of the metaverse: a space designed for users by users, which can satisfy whomever, whatever, however, wherever and whenever [34].

However, in the RSL we find authors who consider that the conception of a true metaverse, in the sense of a digital universe parallel to our analogic world (where the participants can engage in social, economic, artistic or leisure activities beyond just videogames), has yet to be created and is pending the development of the technologies that would make it possible [35]. That’s why numerous companies, such as Meta, Microsoft, Epic Games, and Google, are working on and investing in crucial emerging technologies for the metaverse, such as virtual reality headsets, augmented reality sensors, and blockchain [36]. In any event, the challenges presented by the metaverse, its technology, and its prospective evolution remain largely unknown [37]. This pertains equally to the physical and psychological well-being of both individuals and collectives [38].

The RSL indicates that there are three terms to refer directly to tourism activity in the metaverse: “metaverse tourism” [21], “metaversal tourism” [39] or a more indirect option, “Metaleisure” [40]. The metaverse, when associated with tourism, uses physical reality in combination with mixed reality (MR), with the latter consisting of augmented reality (AR) and virtual reality (VR). So far, the more extensively used terms in the field of tourism research is virtual tourism [3,41], also referred to as “cloud tourism”, which uses both VR and AR technologies, as well as live video streaming [3].

Finally, related to the metaverse concept, a substantial body of evidence suggests that the contactless culture driven by the COVID-19 pandemic [5,6] has promoted the development of the metaverse and, concurrently, has spurred research into the enabling virtual technology of the metaverse [37,42,43]. More specifically, some references delve into its impact on financial markets and the use of NFTs and cryptocurrencies for payment [44], the digital economy [37], or virtual museums [42].

### Statistics on tourism in the metaverse

Through the SLR and complementary research, information is gathered regarding the impact of the metaverse on the tourism sector.

The metaverse is so new that the earliest statistics and estimates date back to 2022. It is predicted that income from a single metaverse performance, such as the Travis Scott concert, would amount to at least $1 million, with a total of $20 million [44]. According to the International Congress and Convention Association [45] the market share for virtual and hybrid gatherings has doubled since 2020. In addition, 61% of presenters, while acknowledging the importance of on-site events, believe that there is a push toward hybrid (on-site and online) events [46]. Thomas Cook, as part of its “Try Before You Fly” campaign, produced a variety of immersive 360º VR contents lasting 5 minutes each, with the goal of presenting New York as a destination. These views allowed the agency to increase reservations for excursions to New York by 190% [47,48]. The figures from Kang’s study [49] also confirm the effectiveness of VR for the tourism sector. VR devices (head-mounted displays (HMD) had a 47% greater telepresence than video and promoted engagement, thus increasing the client’s desire to purchase by 75%. In 2019, 20% of potential tourists expressed interest in VR devices in order to receive travel-related content [50]. In 2021, 9.54 million shipments of augmented reality (AR) and virtual reality (VR) helmets were recorded. In 2022, it is expected that the AR/VR headset shipments to consumers will amount to 13.24 million units [51].

### Most studied technologies for tourism marketing in metaverse

**Fig 3:**
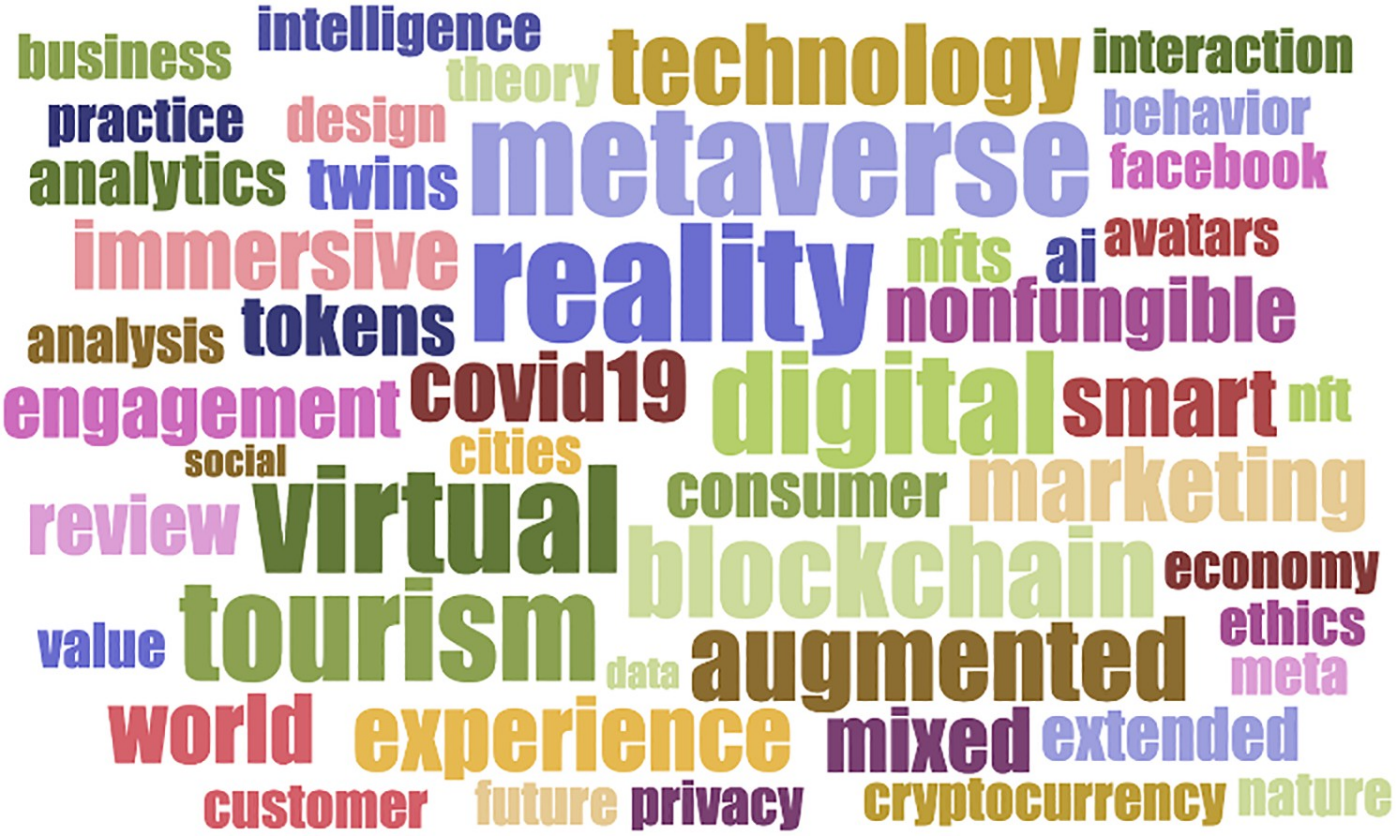
The 50 most frequently repeated keywords in the reports included (n = 86).

The word cloud generated from the 86 articles analyzed in the SLR (Fig 3) serves to illustrate the central technologies and systems involved in the development of tourism marketing in the metaverse.

**Fig 4:**
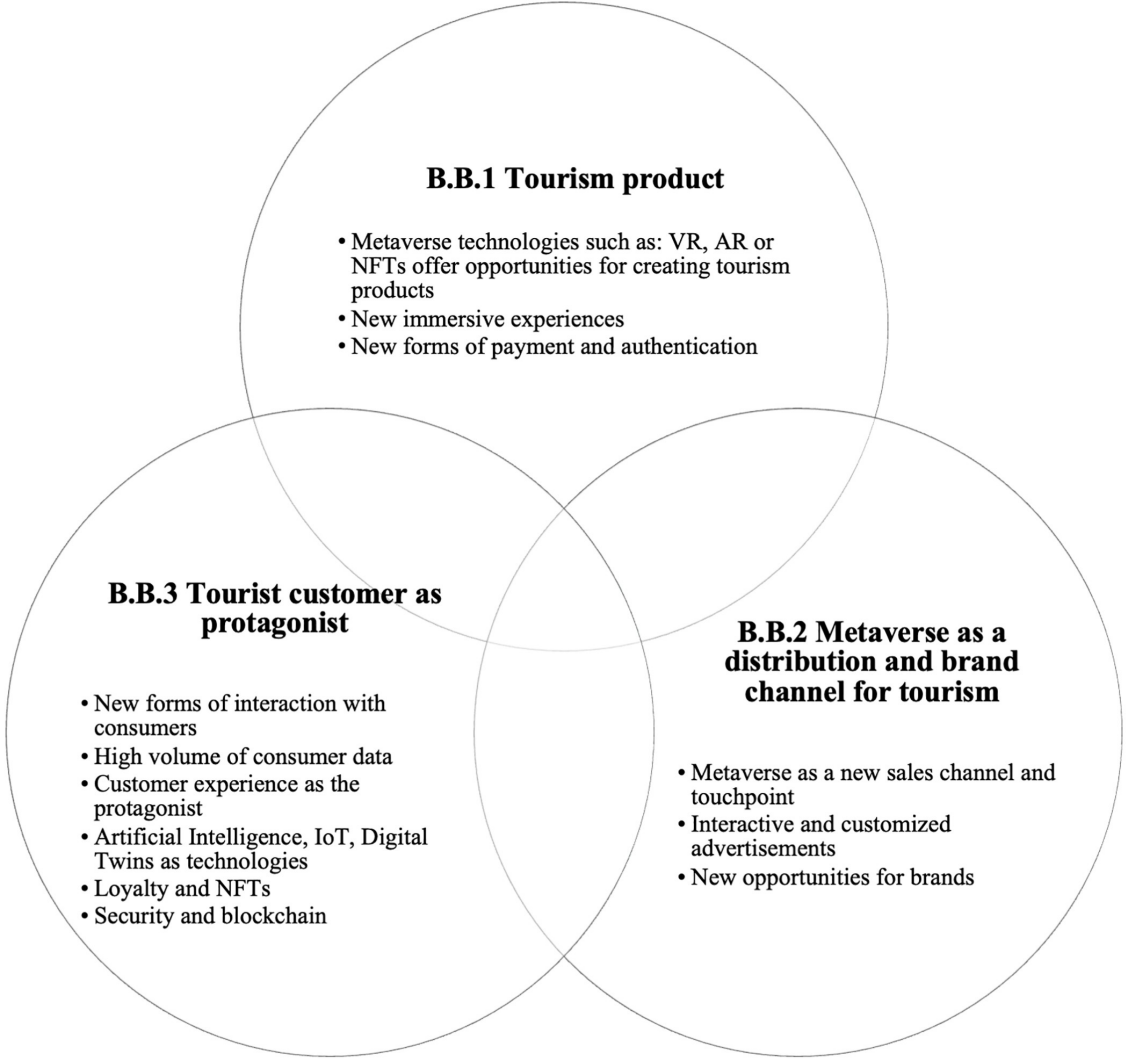
Building blocks of tourism marketing in the metaverse.

RSL reveals the core technologies, systems, and applications associated with tourism marketing, including but not limited to tokens (NFTs), blockchain, virtual reality, augmented reality, mixed reality, AI, and digital twins. Fig 4 shows the results of a tag cloud analysis of 86 articles (based on their keywords). In addition, Internet of Things (IoT), gamification and new payment systems such as cryptocurrencies are also detected as predominant themes (outside the keywords).

#### Blockchain technology and Non-Fungible Tokens (NFT)

The findings from the existing literature have shown that digital technologies play a crucial role in gaining a competitive edge in marketing [52]. Specifically, the application of NFT technology revolutionizes the way in which the content is created, marketed, exchanged, stored, and authenticated, both for the content creators themselves and for their fans.

Non-fungible tokens (NFTs) are transferable rights to digital assets, such as art, collectables, music or game elements. This phenomenon and its markets have grown significantly since early 2021 [53]. NFTs are uniquely certified with blockchain (a set of technologies that make it possible to keep a secure, decentralized, synchronized and distributed register of digital operations without the need for third-party intermediation) authentication [54,55]). There is even mention of a new type of marketing, “NFT marketing”, which is focused on the creation, promotion and strategic use of NFTs to achieve marketing objectives [56].

Fungible goods, such as money or commercial goods, can be exchanged for the same type of goods. On the contrary, non-fungible items cannot be exchanged for a similar product because their value exceeds the real value of the material [53]. NFTs can include the offer of products or services of either a digital or physical nature, with markets for their sale, such as OpenSea and Rarible. Authors like [57] introduced a transformative category in NFTs known as Dynamic Non-Fungible Tokens (dNFTs), representing a pioneering advancement within the NFT landscape. These dNFTs extend the scope of NFTs to include a broad spectrum of products and services, encompassing both digital and physical offerings.

For the travel sector, offering NFT-based services to passengers by travel companies is a savvy approach that allows these companies to track passengers, gather and analyze customer data, and enhance service levels [58].

Cryptocurrencies are used to make purchases, primarily in Ethereum (ETH), as a payment and negotiation option, which demonstrates a close relationship between the cryptocurrency market and the NFT market [59]. Cryptocurrencies are modifying the very nature of how travelers use and manage payment systems [60]. The adoption of new payment methods by companies in the tourism sector poses a series of clear advantages: differentiation from companies that do not accept them, an increased conversion rate related to offering more alternatives for reservations and the security offered by collecting non-reimbursable fees [61].

#### VR, AR and MR technology

The literature is clear that virtual reality is making progressive advancements and becoming increasingly adopted. It is widely acknowledged in the SLR that virtual reality (VR) technologies are being used by millions of people. This is especially true after the outbreak of Covid-19, as VR platforms like VR Chat, Facebook Horizon, and Rec Room experienced exponential growth due to its secure and attractive way of connecting with others, when travel and social gatherings were heavily restricted. Through virtual reality, users can experience interacting with others in seemingly infinite worlds, creating their own avatars, and enjoying a social atmosphere comparable to reality [62]. VR creates a completely digital environment that is cut off from the outside world [63], in which the user (or their avatar) navigates through a virtual environment [49]. Authors, such as Jaung [64] in the field of natural sciences, explain how metaverse technologies, including VR, provide a new way of interacting with nature through immersive three-dimensional virtual worlds.

In terms of marketing, VR can be employed by specialists to co-create value with consumers and promote consumer-brand engagement [65].

Augmented reality (AR) is another essential technology for metaverse tourism activities. AR has emerged as an innovative communication device that adds virtual information to a user’s real-world environment [63]. It enhances the real-world atmosphere by providing context-sensitive data [66], such as numbers, letters, symbols, audio, video and graphics [67]. AR and VR are the most prominent examples of immersive technologies [68]. Mixed reality (MR) intertwines real and virtual worlds [63], while extended reality (XR) serves as an umbrella term encompassing previous technologies [69].

#### Artificial intelligence (AI) and digital twins

Other common themes observed in the SLR included artificial intelligence and digital twins. AI can offer highly precise information to assist an organization in making better decisions based on collected data [70]. The global pandemic has spurred many organizations to accelerate investments in AI to optimize production capacity, logistics, and customer management [71]. An example of this is the use of digital twins. These are digital replicas of physical objects, processes, or services which allow the collection of data to create simulations that model, test, and predict the performance of a product, process, or service in the real world [72–74]. Digital twins can be developed for objects, buildings, services, systems, and even cities. By combining big data, Internet of Things (IoT) solutions, AI, and data analysis, digital twins allow us to analyze data and simulate potential future scenarios.

### Practical cases of tourism marketing in the metaverse

Most of the articles analyzed in this research provide examples that help to understand how metaverse technologies are incorporated into the tourism marketing. While the metaverse (or metaverses) is still in development [75], the tourism industry is already using its technologies. Those involved, such as hotels, restaurants, transport, leisure activities, and destinations, “tangibilize” their services and offer immersive experiences to the public [21]. Table 2 offers a summary of the examples presented in the SLR and supplementary research, regarding the implementation of metaverse technologies, systems and applications in the tourism marketing, covering destinations, hotels, restaurants, transportation, and leisure/cultural activities (e.g., concerts, theatre and museums).

**Table 2:** Examples of applying metaverse technologies and systems in the tourism marketing.

Hospitality services	Metaverse technologies, systems, and applications	Building Blocks examples	Examples found in the SLR and complementary research
**Destinations**	VR	B.B.1	Subscription to National Geographic VR allows users to use Oculus VR equipment to virtually sail through icebergs in Antarctica and explore the hidden treasures of Machu Picchu [20]
	VR	B.B.1	The view of the Grand Canal from the Rialto bridge in Venice could be shown virtually through VR [76]
	Blockchain-NFT	B.B.1	NFTs could also be used as an alternative means to preserve a destination [77]. Specifically, the proposal by Mofokeng & Fatima [78] is focused on preserving wildlife. The authors state that blockchain technology, through NFTs, makes it possible to clone wildlife species, with all their characteristics, to preserve and market them. The non-fungible aspect provided by blockchain technology could guarantee that conservation areas that are home to species in danger of extinction can benefit from the sale of their NFT. This would provide an alternative model of income for the preservation and conservation of these territories.
	AR Gamification	B.B.1- B.B.2	The city of Santa Monica is using AR to attract more people to its shops and events in the city center. Through the FlickPlay application, users have access to an interactive map of tokens spread out around the city. The objective is that through a gamified experience, both residents and tourists can visit the city and walk up to shops and shop windows. In return, the city benefits by promoting local businesses, which are important pieces for bringing income into the city [72].
	AR	B.B.1-B.B.2	In Pompeii (Italy), tour guides offer the opportunity to use augmented reality glasses (AR Glasses) to take an innovative and immersive tour, as an element that sets it apart from the competition. AR technology creates holograms that are superimposed over the existing ruins, showing what the temples, houses, squares, theatres and the most important buildings were like before the volcanic eruption [79].
	AR-IoT	B.B.1	The combination of AR and IoT applications can provide travellers with information about the status of flights and luggage, assistance in unfamiliar tourist destinations (using interactive applications with subway and bus maps, museum guides or taxi reservation services, using their current location [67].
	VR	B.B.1	At the Google IO 2022 conference, Google presented a prototype of their new version of Google Glasses. This device has a microphone that records the speaker’s voice, transcribes it and translates it into another language in real time, projecting the text onto the lens of the glasses. This product will allow everything from simultaneous translations at the company level to a fully touristic use that facilitates communication by tourists at the destination [80].
	Gamification	B.B.2	Thailand Tourism has promoted its country brand with a gamification strategy by partnering with video game companies and creating “Smiled Land Thailand”, which was designed to show off the tourist attractions in the country, promoting the destination and positioning the brand [81,82].
	Digital Twins, AI, IoT	B.B.3	The German city of Herrenberg has implemented its own digital twin that contributes to sustainable urban planning (air quality, traffic, pedestrian flows and even emotions of residents) ([83]).
**Hotels**	Blockchain-NFT	B.B.1-B.B.2	New York’s Nomo Soho Hotel sells a tourism package in NFT format on OpenSea [84].
	Cryptocurrencies	B.B.1	Examples of hotels in Spain that have been pioneers in accepting payment with cryptocurrencies are the Bécquer and Kivir hotels in Seville [85]
**Restaurants**	AR	B.B.1	Using a QR code scanner to see a restaurant menu using AR has become commonplace following the pandemic [66].
	VR Gamification	B.B.2	The fast food chain Wendy’s Co. opened a virtual restaurant in Horizon Worlds, the Meta virtual reality game. Players cannot buy food (either virtually or physically); they can only play basketball in a place located near the restaurant, as if it were a product placement strategy. Despite this, Wendy’s Co. understands that Horizons Worlds provides them with an authentic and different way to earn brand recognition [86].
	Gamification	B.B.3	As an example of a customer loyalty program, we have Chipotle Mexican Grill, Inc. The restaurant brand got onto the Roblox Corp. gaming platform and, through a gamification strategy, offered coupons that could be exchanged for real food at Chipotle restaurants [86].
	Blockchain-NFT	B.B.3	The Californian winery Yao Family Wines has auctioned off their ’The Chop Drop’ wine, accompanied by a limited edition NFT. This makes it the first winery in the world to offer an auctioned wine together a digital NFT collectable. Each NFT represents a specific limited edition numbered bottle [87].
**Means of transport**	Blockchain-NFT	B.B.3	In late February 2020, Emirates Airlines implemented a customer loyalty system blockchain, which revolved around NFT experiences. The objective was to reduce operating costs, improve the quality of customer service and reduce the financial liabilities related to large-scale loyalty programs [88,89]. The airline touted the NFTs as rewards, giving shape to a new type of loyalty program. For example, once a customer reaches a certain number of flight tickets, he or she earns an NFT [90].
**Leisure and cultural activities: concerts, theatre and museums**	Gamification	B.B.1 -B.B.2	Travis Scott, a U.S. hip-hop artist, gave a concert in the metaverse on the Fortnite platform in 2020, with more than 12 million viewers in real time. The same occurred with Ariana Grande, who managed to draw 78 million people on the same platform [44,91].
	Blockchain-NFT	B.B.1 -B.B.3	Customers purchasing the NFT album by the U.S. rock band the *Kings of Leon* had access to unreleased music, exclusive works of visual art and backstage passes to the shows [56].
	VR	B.B.1	Theatre companies and other creative projects, such as the Royal Shakespeare Company, Double Eye Productions or The Fourth Wall VR, have produced innovative theatre experiences in VR [62].
	AR-VR	B.B.1	In the multimedia Van Gogh Alive exhibition, exhibited in 130 countries across six continents, the painter’s work is brought to life through projections that combine light, colour and music to give the viewer the sensation of being part of the paintings [76].
	Blockchain-NFT	B.B.3	The Royal Museum of Fine Arts Antwerp (KMSKA), meanwhile, has introduced a system of Art Security Tokens to make masterpieces accessible to the public. The museum proposes to build a community, maintaining a close relationship with investors after they purchase the tokens. For example, KMSKA will exclusively invite all token holders to the museum to see the piece of art in which they have invested [92] and the Kharkiv Art Museum in Ukraine has launched a new non-fungible token (NFT) collection with Binance to support Ukrainian cultural heritage.
	Gamification	B.B.1	On the ZEPETO platform, Naver Z created the virtual Museum Renaissance experience, which unlike physical museums, makes it possible to interact with work of art (e.g., to take photos or videos with the works of art, to sit in the museum or even run in it [4]).

### Metaverse building blocks

In the SLR, two articles ([6,22]) were found that discussed the fundations and the building blocks of marketing in the metaverse. These early contributions shed light on the beginning stages of marketing activities within the metaverse.

Dwivedi et al. [6] describe the foundations of metaverse marketing, based on five key elements: product, branding, distribution channels, consumer interaction, and customer information. NFTs allow for unique virtual products, VR and AR offer new branding opportunities, AI agents provide personalized consumer interactions, and the metaverse itself is a treasure trove of consumer information.

Hollensen, Kotler & Opresnik [22], on the other hand, explain the nine building blocks of marketing in the metaverse, including hardware, networking, computers, virtual platforms, interchange standards and tools, payments, content, services and assets, consumer and business behaviours. Hardware such as VR headsets, mobile phones and haptic gloves provides access to the metaverse, networking ensures data transmission and reliability, computing power providing the necessary resources for metaverse to function properly, virtual platforms enable users to interact with the metaverse, interchange standards allow for interoperability, payments (cryptocurrencies and digital currencies) cover purchases and transfer of money, content and services provide experiences, and consumer and business behaviours are shaped by the metaverse.

Outside the SLR, [93] outlines the key building blocks and future challenges of the metaverse as: 1) ethical, regulatory, governance, security, and privacy challenges; 2) an ecosystem including enterprise and consumer use cases, content creation, virtual economy, and avatars; and 3) the underlying technologies, such as extended reality (VR/AR), user interfaces, AR, blockchain, and edge computing.

Furthermore, outside of the RSL, [94] mention the major technological building blocks: networks, computing, 3D modeling, IoT, AI, blockchain, XR, and interface devices, each of which is explored in brief.

Buhalis, Leung & Lin [23] emphasize that the development and success of tourism in the metaverse is dependent upon certain key building blocks: 1) networking infrastructure (hardware devices, software applications, and network services), 2) enabling devices (such as MR/VR headsets and environment rendering devices), 3) empowering platforms or virtual worlds (with high-fidelity graphics and immersive experiences), and 4) technology-ready users (there is an increasing demand for users who are willing to engage in the metaverse).

This search revealed a gap in the literature regarding the building blocks of tourism marketing within the metaverse.

### A proposal of the building blocks of tourism marketing in the metaverse

This article seeks to fill the gap by proposing the foundations of tourism marketing in the metaverse. For this purpose, [6] and [22] are taken as a starting point, which is then supplemented by the insights drawn from the SLR and complementary research. The information in this section can be completed with the practical examples presented in Table 2.

- B.B.1. **Tourism product**. The classic definition of the tourism product, as a combination of elements (natural, cultural, anthropogenic, facilities, services, attractions and activities) that represents the essence of a destination’s marketing plan and generates a comprehensive tourism experience for potential clients that is marketed through distribution channels which set the price and have a life cycle [95], expands its marketing opportunities in the metaverse. Drawing from the SLR and complementary research, three key insights are identified regarding tourism products adapting to metaverse technologies.
  - Examples found in 2022 mainly focused on the use of NFTs (blockchain technology) and virtual reality (VR), augmented reality (AR), and mixed reality (MR) (see Table 2).
  - Virtual Reality (VR) is enabling immersive experiences that can create avatars that embody a new traveller identity for the tourism industry. This allows them to virtually explore destinations they have visited as well as new ones and engage in creative fantasy experiences in the metaverse [21]. Also, thanks to gamification, new tourist products can be created, with greater interaction even than the real tourist product, as is the case of the virtual Museum Renaissance (see Table 2).
  - Companies in the tourism sector are increasingly adopting new payment methods to differentiate themselves from their competitors, improve customer conversion rates, and gain the security of collecting non-refundable fees [61]. For instance, the Nomo Soho Hotel in New York offers tourism packages in NFT format for sale on the OpenSea token marketplace through the Ethereum cryptocurrency [84]. This serves as another example of a novel type of tourism product.
- B.B.2. **The metaverse as a distribution and branding channel for tourism**.
  We have just seen that the metaverse is a platform that makes it possible to create tourism products that are different from the traditional ones.
  - But it should also be understood as a new sales channel, a touchpoint so brands can communicate with their customers [96]; a way to offer innovative omnichannel experiences that allow brands to position themselves in the minds of consumers and open up new markets. It is possible, thanks to metaverse, for brands to penetrate a digital worldwide market, through virtual communication, digital branding, and online marketing [97].
  - Brands have the opportunity to adopt totally new ways of interacting with users in the metaverse and launch fully customized offers through immersive virtual spaces [22,98]. Emerging specialties such as avatar marketing are gaining traction as a brand reinforcement strategy [99]. Also, advertisements for a brand will be interactive and customized. AR and VR technologies will allow users try out the product or service before they buy it [97], through strategies like “try before you buy” [3].
  - NFTs also play a crucial role in brand positioning in the metaverse. Promoting storytelling and collectable assets in token format, even prior to the product launch (which can be digital or physical), will create interest in the product and the brand being marketed, as well as a new flow of income even before the product is available for sale. In the years to come, NFT could be the central touchpoint between brands and their consumers [100].
  - From the distribution point of view, NFT can break down the barriers between the physical and virtual worlds in a way that is similar to how modern omnichannel marketing systems integrate traditional distribution channels with online shopping. NFTs eliminate the barrier of the intermediary, thanks to blockchain technology. AI will be used to automate smart contracts, decentralized accounting books and other blockchain technologies to allow virtual transactions. In this terrain free of intermediaries or control, there will be a need to establish the rules of the game that make it possible to comply with the stipulated ethical codes [101].
- B.B. 3. **Tourist customer as protagonist**. This involves enabling consumer interaction, customer experience, and obtaining customer information in the metaverse.
  - Metaverse technologies allow for new and immersive interaction with customers, as well as providing useful data on customers beyond social media [6]. Enriched data about buyers and analytical capacities help to define customer profile and therefore to improve the sopping experience in virtual environments, promoting brand loyalty in the worlds of the metaverse [98,102].
  - Analytic marketing helps companies optimize campaigns, segment markets, reduce costs [103] and make better decisions [104]. Artificial Intelligence (AI) is being used to create digital twins that can provide data to simulate scenarios and further develop customer experience [105]. Blockchain technology is also being employed to promote ethical marketing strategies such as loyalty programs, traceable online advertising, brand transparency in online markets [86,89] or to claim ownership of original digital works and build loyalty [92].

The Fig 4 shows a graphical summary of Building Blocks of tourism marketing in the metaverse.

## Discussions, conclusions and future research directions

Research into the topic of the metaverse, especially within the tourism sector, addresses an important priority because the impact of the metaverse in tourism marketing is still novel. The tourism industry is an interesting and relevant field of study due to its influence on the economy, society, culture, and environmental aspects of nations worldwide.

Screening of the SLR reveals that topics related to the metaverse evolve over time. Articles published prior to 2020 mainly focus on topics like Second Life, virtual world, and 3D, while more recent articles discuss concepts such as metaverse, blockchain, XR, AR, VR, Second Life, AI, virtual world, IoT, and NFTs. The majority of publications come from European universities, with the United Kingdom (12) and Germany (4) leading the way in research. Asia is also a major source of research, with South Korea (8) and China (7) being notable contributors. Finally, in America, United States produced the most number of publications, with 11 articles present in the literature review. Overall, these findings indicate that research into the metaverse and its implications for the tourism sector is an emergent research topic, with universities in European and North American countries leading the scientific research in this field, alongside Asian countries like South Korea and China, possibly driven by their growing interest in technological advancement.

Connecting the topic with existing theory, this research is presented as the first to conduct a SLR on tourism marketing in metaverse. The technologies that have been most thoroughly studied at the confluence of marketing, tourism, and the metaverse are: AI, virtual reality, augmented reality, mixed reality, blockchain, tokens (NFTs) and digital twins. Dwivedi et al. [6] and Hollensen, Kotler & Opresnik [22] explore the fundamental concepts and elements of marketing within the metaverse, whereas Buhalis, Leung & Lin [23] delve into the components of tourism in the metaverse. However, there is an absence of references pertaining to the building blocks of tourism marketing within the metaverse.

Given the embryonic stage of metaverse development and the limited knowledge surrounding its impact on society [106], an analysis of its evolution and adaptation to different sectors is needed to ensure it is established securely. The metaverse has already begun to transform the way people buy, work, socialize, and entertain, particularly for young early adopters. This article proposes the tourism industry as a case study to investigate marketing in the metaverse. In recent years, technology and creativity have been the driving forces behind the sector’s development. Creative industries are vital, accounting for 3% of the world’s GDP, and the current pandemic has caused them to grow and become more digitalized with the help of advanced technologies. Results from this research can be applied to other fields in the creative industries and will help to create knowledge around metaverse marketing.

Consequently, our research seeks to establish the building blocks of tourism marketing in the metaverse, by combining insights from the literature review with complimentary research, based on: the tourism product; the metaverse as a distribution and branding channel for tourism; tourist customer as protagonist. The research results show how the tourism product changes with the metaverse. Tourism products can be adapted to the metaverse with digital offerings, such as blockchain technology (e.g., NFTs) and virtual reality, and gamification allowing for creative fantasy experiences. The metaverse can be used as a sales channel and touchpoint between brands and their customers, with technologies such as AR, VR and NFTs enabling immersive experiences and personalized offers. Additionally, AI and blockchain can be used to create customer interaction, experience, and collect customer data in the metaverse. Tourism sector can benefit from the use of blockchain technology to claim ownership of digital works and build loyalty.

The systematic literature review points toward prospective research directions. Investigating tourism marketing in the metaverse should encompass several key areas:

- Evaluating customer engagement and virtual experiences [98]. For example, the introduction of smart glasses by Ray-Ban, equipped with advanced features like a 5MP camera, three microphones, speakers, Bluetooth, and Wi-Fi capabilities, has the potential to transform the way travel experiences are captured and shared. This innovation also impacts the role of social media in sharing tourism experiences [107].
- To keep customers motivated and engaged in the metaverse, gamification programs are expected to play a crucial role [108].
- The use of blockchain technology can significantly contribute to creating a secure, decentralized, synchronized, and distributed record of digital transactions, eliminating the need for third-party intermediaries. This has the potential to revolutionize various aspects of the metaverse [54,55], including the potential of blockchain integration with the Internet of Things [109].
- Research is also needed to explore the monetization of products in the metaverse, including the adoption of NFTs [53] the digital payment systems (DPS 2.0) [103,110]. It is essential to investigate which payment methods will be used in the metaverse and what new VR experiences will enable future tourists to experience [21,111].
- The use of metaverses introduces new ways of business-to-consumer interaction that enable the simulation of the physical world in the virtual world [112]. Research should explore how metaverse distribution channels can become replacements for physical channels.
- Additionally, research should investigate the potential for experience-based strategies instead of price strategies, as well as how intermediaries may be redefined in the metaverse.
- It will also be necessary to consider what happens to companies and tourist destinations that don’t opt to use metaverse technologies.
- The new profiles of marketing professionals and policy development that regulates commercial activity in the metaverse necessitates skills and knowledge focused on technology, predictive analytics, innovative strategies, and new mechanisms [113]. Tourism marketing professionals will need to adapt to the demands of the metaverse, as well as their corresponding training, skills and abilities.
- Finally, research is needed to determine the ethical challenges associated with the development of virtual reality [114] as well as measures to protect customer data in the metaverse and how users can control, share, or monetize their data online [70,115].

## Limitations

Due to its novelty, the research presents certain limitations in terms of the number of references analyzed. In addition, the fast evolution of both technology and publications on the metaverse means that is will be necessary to update the number of published articles on a monthly basis.
